# Supplementary material for: Quality and variation of care for chronic kidney disease in Swiss general practice: A retrospective database study
Source: PLoS One. 2022 Aug 11;17(8):e0272662. doi: 10.1371/journal.pone.0272662 (PMC9371276; doi:10.1371/journal.pone.0272662)
Supplement: S1 Table — Criteria were defined using International Classification of Primary Care (ICPC-2) codes, Anatomical Therapeutic Chemical (ATC) codes, vital parameters, and laboratory values. (PDF) [file pone.0272662.s001.pdf]

S1 Table. Operationalized criteria for identification of conditions and events. Criteria were defined using International Classification of Primary Care (ICPC-2) codes, Anatomical Therapeutic Chemical (ATC) codes, vital parameters, and laboratory values.

**Characteristic**	**Description**
Diabetes	At least one of the following:<br>– ICPC-2 codes T89/T90;<br>– active prescription of ATC codes in class A10 (drugs used in diabetes) with exception of class A10BJ (glucagon-like peptide-1 analogues);<br>– two consecutive measurements of glycated hemoglobin (according to the *National Glycohemoglobin Standardization* Program norm [1]) of at least 6.5% [2].
Hypertension	At least one of the following:<br>– ICPC-2 codes K85/K86/K87;<br>– two consecutive blood pressure measurements with levels above 140 (systolic) and/or 90 (diastolic) mmHg [3];<br>– active prescription of ATC codes classified into the pharmaceutical cost group “Hypertonie” as outlined in [4].
Established cardiovascular disease	At least one of the following:<br>– ICPC-2 codes K74–76/K82/K89–92;<br>– active prescription of ATC codes in classes B01AC04 (clopidogrel), B01AC06 (acetylsalicylic acid), B01AC07 (dipyridamole), B01AC22 (prasugrel), B01AC24 (ticagrelor), B01AC25 (cangrelor), B01AC26 (vorapaxar)
Renin-angiotensin-aldosterone system inhibitor prescription	Active prescription of ATC codes in class C09 (agents acting on the renin-angiotensin system).
Non-steroidal anti-inflammatory drug prescription (except acetylsalicylic acid)	Active prescription of ATC codes in class M01A (anti-inflammatory and antirheumatic products, non-steroids).

References

1. Little RR, Rohlfing C, Sacks DB. The National Glycohemoglobin Standardization Program: Over 20 Years of Improving Hemoglobin A(1c) Measurement. Clin Chem. 2019;65(7):839-48. Epub 2018/12/05. doi: 10.1373/clinchem.2018.296962. PubMed PMID: 30518660.

2. Rydén L, Grant PJ, Anker SD, Berne C, Cosentino F, Danchin N, et al. ESC Guidelines on diabetes, pre-diabetes, and cardiovascular diseases developed in collaboration with the EASD: the Task Force on diabetes, pre-diabetes, and cardiovascular diseases of the European Society of Cardiology (ESC) and developed in collaboration with the European Association for the Study of Diabetes (EASD). Eur Heart J. 2013;34(39):3035-87. Epub 2013/09/03. doi: 10.1093/eurheartj/eht108. PubMed PMID: 23996285.

3. Mancia, G., et al., 2013 ESH/ESC guidelines for the management of arterial hypertension: the Task Force for the Management of Arterial Hypertension of the European Society of Hypertension (ESH) and of the European Society of Cardiology (ESC). Eur Heart J. 2013;34(28):2159-219. Epub 2013/06/19. doi: 10.1093/eurheartj/eht151. PubMed PMID: 23771844.

4. Federal Office of Public Health (Health and Accident Insurance Directorate Premiums, Solvency Oversight Section). Liste der pharmazeutischen Kostengruppen (PCG-Liste). Bern, Switzerland: 2021 [cited 2021 Feb 20]. Available from: https://www.bag.admin.ch/bag/en/home/versicherungen/krankenversicherung/krankenversicherung-versicherer-aufsicht/risikoausgleich.html.
