# Supplementary material for: Quality and variation of care for chronic kidney disease in Swiss general practice: A retrospective database study
Source: PLoS One. 2022 Aug 11;17(8):e0272662. doi: 10.1371/journal.pone.0272662 (PMC9371276; doi:10.1371/journal.pone.0272662)
Supplement: S2 Table — “Full model” denotes the regression model including all predictors, while “Null model” denotes the model where demographic characteristics of general practitioners (GPs) were omitted. Abbreviations: CI, confidence interval; ICC, intraclass correlation coefficient; OR, odds ratio; QI, quality indicator. (PDF) [file pone.0272662.s002.pdf]

S2 Table. Determinants of quality indicator achievement in the category *Assessment*. "Full model" denotes the regression model including all predictors, while "Null model" denotes the model where demographic characteristics of general practitioners (GPs) were omitted. Abbreviations: CI, confidence interval; ICC, intraclass correlation coefficient; OR, odds ratio; QI, quality indicator.

	QI 1		QI 2		QI 3	
**Full model**	**OR (95 % CI)**	***p*-value**	**OR (95 % CI)**	***p*-value**	**OR (95 % CI)**	***p*-value**
Intercept	1.91 (1.23–2.99)	0.004*	1.37 (0.97–1.93)	0.07	1.71 (0.99–2.96)	0.05
Male patient	1.14 (1.04–1.25)	0.01*	1.11 (1.05–1.16)	< 0.001*	1.05 (0.96–1.14)	0.27
Patient age: 40–59 years (reference: < 40)	2.26 (1.84–2.78)	< 0.001*	1.65 (1.45–1.88)	< 0.001*	1.59 (1.08–2.33)	0.02*
Patient age: 60–79 years (reference: < 40)	3.62 (2.96–4.43)	< 0.001*	2.71 (2.39–3.08)	< 0.001*	2.42 (1.66–3.54)	< 0.001*
Patient age: ≥ 80 years (reference: < 40)	4.67 (3.70–5.89)	< 0.001*	3.54 (3.09–4.06)	< 0.001*	2.85 (1.94–4.19)	< 0.001*
Male GP	0.67 (0.47–0.94)	0.02*	0.74 (0.57–0.97)	0.03*	0.80 (0.57–1.11)	0.18
GP age: 45–59 (reference: < 45)	0.90 (0.64–1.27)	0.56	0.89 (0.68–1.17)	0.41	0.83 (0.59–1.16)	0.28
GP age: ≥ 60 (reference: < 45)	0.73 (0.43–1.22)	0.23	0.84 (0.55–1.27)	0.40	0.82 (0.49–1.38)	0.47
Urban practice location	1.12 (0.78–1.61)	0.54	1.07 (0.80–1.44)	0.64	1.11 (0.78–1.60)	0.56
GP-level group variance, ICC	1.46, 0.31		1.01, 0.24		1.30, 0.28	
**Null model**						
Intercept	1.39 (0.97–1.99)	0.07	1.04 (0.79–1.37)	0.79	1.16 (0.73–1.85)	0.54
Male patient	1.13 (1.03–1.24)	0.01*	1.12 (1.07–1.17)	< 0.001*	1.06 (0.98–1.14)	0.16
Patient age in years (reference: < 40)						
40–59	2.35 (1.93–2.88)	< 0.001*	1.66 (1.46–1.88)	< 0.001*	1.88 (1.30–2.71)	< 0.001*
60–79	3.68 (3.02–4.48)	< 0.001*	2.74 (2.42–3.10)	< 0.001*	2.84 (1.98–4.07)	< 0.001*
≥ 80	4.64 (3.71–5.82)	< 0.001*	3.53 (3.09–4.03)	< 0.001*	3.26 (2.26–4.69)	< 0.001*
Urban practice location	1.07 (0.75–1.53)	0.70	1.08 (0.81–1.44)	0.62	1.09 (0.77–1.55)	0.63
GP-level group variance, ICC	1.48, 0.31		1.03, 0.24		1.30, 0.28	

*Statistically significant at level 0.05.
