# Supplementary material for: Quality and variation of care for chronic kidney disease in Swiss general practice: A retrospective database study
Source: PLoS One. 2022 Aug 11;17(8):e0272662. doi: 10.1371/journal.pone.0272662 (PMC9371276; doi:10.1371/journal.pone.0272662)
Supplement: S3 Table — “Full model” denotes the regression model including all predictors, while “Null model” denotes the model where demographic characteristics of general practitioners (GPs) were omitted. Abbreviations: CI, confidence interval; eCVD, established cardiovascular disease; ICC, intraclass correlation coefficient; OR, odds ratio; QI, quality indicator. (PDF) [file pone.0272662.s003.pdf]

S3 Table. Determinants of quality indicator achievement in the category *Monitoring*. "Full model" denotes the regression model including all predictors, while "Null model" denotes the model where demographic characteristics of general practitioners (GPs) were omitted. Abbreviations: CI, confidence interval; eCVD, established cardiovascular disease; ICC, intraclass correlation coefficient; OR, odds ratio; QI, quality indicator.

	QI 4		QI 5		QI 6		QI 7		QI 8	
**Full model**	**OR (95 % CI)**	***p*-value**	**OR (95 % CI)**	***p*-value**	**OR (95 % CI)**	***p*-value**	**OR (95 % CI)**	***p*-value**	**OR (95 % CI)**	**p-value**
Intercept	2.01 (1.58–2.57)	< 0.001*	0.17 (0.10–0.30)	< 0.001*	0.60 (0.44–0.82)	0.001*	0.66 (0.48–0.91)	0.01*	0.42 (0.26–0.66)	< 0.001*
Diabetes	1.60 (1.45–1.76)	< 0.001*	3.76 (3.17–4.45)	< 0.001*	1.68 (1.52–1.85)	< 0.001*	1.55 (1.39–1.73)	< 0.001*	1.20 (1.00–1.44)	0.05*
Hypertension	1.30 (1.19–1.41)	< 0.001*	1.17 (0.98–1.39)	0.08	1.58 (1.45–1.73)	< 0.001*	1.49 (1.34–1.64)	< 0.001*	1.31 (1.10–1.56)	0.002*
eCVD	1.37 (1.24–1.51)	< 0.001*	0.90 (0.74–1.10)	0.31	1.25 (1.14–1.38)	< 0.001*	1.25 (1.12–1.40)	< 0.001*	1.03 (0.85–1.25)	0.75
Male patient	1.10 (1.01–1.19)	0.02*	1.51 (1.29–1.77)	< 0.001*	1.20 (1.11–1.30)	< 0.001*	1.20 (1.09–1.31)	< 0.001*	1.18 (1.00–1.39)	0.04*
Patient age: 65–79 years (reference: < 65)	1.11 (0.96–1.30)	0.16	0.62 (0.49–0.79)	< 0.001*	1.21 (1.02–1.43)	0.02*	1.27 (1.05–1.53)	0.02*	1.46 (1.04–2.06)	0.03*
Patient age: ≥ 80 years (reference: < 65)	1.03 (0.89–1.20)	0.65	0.22 (0.17–0.28)	< 0.001*	1.03 (0.88–1.21)	0.72	1.12 (0.93–1.35)	0.24	1.04 (0.75–1.46)	0.80
Male GP	0.84 (0.71–0.99)	0.03*	0.86 (0.59–1.26)	0.44	1.00 (0.81–1.24)	0.99	0.84 (0.68–1.05)	0.13	0.87 (0.67–1.14)	0.31
GP age: 45–59 years (reference: < 45)	0.80 (0.68–0.95)	0.01*	1.69 (1.13–2.53)	0.01*	0.99 (0.79–1.24)	0.95	0.99 (0.79–1.24)	0.91	1.14 (0.86–1.50)	0.36
GP age: ≥ 60 years (reference: < 45)	0.79 (0.62–1.01)	0.06	0.82 (0.45–1.51)	0.53	0.80 (0.58–1.10)	0.17	0.72 (0.52–1.01)	0.06	0.99 (0.67–1.48)	0.98
Urban practice location	0.94 (0.80–1.11)	0.49	0.80 (0.53–1.22)	0.30	1.14 (0.91–1.43)	0.25	1.21 (0.96–1.52)	0.11	1.26 (0.96–1.65)	0.10
GP-level group variance, ICC	0.33, 0.09		1.20, 0.27		0.78, 0.19		0.67, 0.17		0.46, 0.12	
**Null model**										
Intercept	1.50 (1.24–1.83)	< 0.001*	0.21 (0.14–0.31)	< 0.001*	0.58 (0.46–0.74)	< 0.001*	0.57 (0.44–0.75)	< 0.001*	0.41 (0.28–0.62)	< 0.001*
Diabetes	1.55 (1.41–1.71)	< 0.001*	3.77 (3.19–4.46)	< 0.001*	1.66 (1.51–1.82)	< 0.001*	1.54 (1.38–1.71)	< 0.001*	1.22 (1.02–1.46)	0.03*
Hypertension	1.29 (1.19–1.41)	< 0.001*	1.17 (0.99–1.39)	0.07	1.55 (1.42–1.69)	< 0.001*	1.49 (1.35–1.65)	< 0.001*	1.32 (1.11–1.57)	0.002*
eCVD	1.35 (1.23–1.49)	< 0.001*	0.92 (0.75–1.11)	0.37	1.26 (1.15–1.38)	< 0.001*	1.26 (1.13–1.41)	< 0.001*	1.03 (0.86–1.24)	0.75
Male patient	1.12 (1.03–1.21)	0.01*	1.50 (1.28–1.75)	< 0.001*	1.21 (1.12–1.31)	< 0.001*	1.19 (1.08–1.30)	< 0.001*	1.17 (0.99–1.37)	0.06
Patient age: 65–79 years (reference: < 65)	1.12 (0.97–1.30)	0.12	0.64 (0.51–0.81)	< 0.001*	1.19 (1.01–1.40)	0.03*	1.25 (1.03–1.51)	0.02*	1.44 (1.02–2.03)	0.04*
Patient age: ≥ 80 years (reference: < 65)	1.05 (0.91–1.22)	0.49	0.23 (0.18–0.29)	< 0.001*	1.00 (0.86–1.18)	0.96	1.10 (0.91–1.32)	0.34	1.05 (0.75–1.46)	0.79
Urban practice location	0.96 (0.82–1.13)	0.65	0.76 (0.50–1.14)	0.19	1.17 (0.94–1.46)	0.15	1.21 (0.96–1.52)	0.11	1.23 (0.94–1.62)	0.13
GP-level group variance, ICC	0.33, 0.09		1.26, 0.28		0.77, 0.19		0.67, 0.17		0.46, 0.12	

*Statistically significant at level 0.05.
