# Supplementary material for: Quality and variation of care for chronic kidney disease in Swiss general practice: A retrospective database study
Source: PLoS One. 2022 Aug 11;17(8):e0272662. doi: 10.1371/journal.pone.0272662 (PMC9371276; doi:10.1371/journal.pone.0272662)
Supplement: S4 Table — “Full model” denotes the regression model including all predictors, while “Null model” denotes the model where demographic characteristics of general practitioners (GPs) were omitted. Abbreviations: CI, confidence interval; eCVD, established cardiovascular disease; ICC, intraclass correlation coefficient; OR, odds ratio; QI, quality indicator. (PDF) [file pone.0272662.s004.pdf]

**S4 Table. Determinants of quality indicator achievement in the category *Medication*.** "Full model" denotes the regression model including all predictors, while "Null model" denotes the model where demographic characteristics of general practitioners (GPs) were omitted. Abbreviations: CI, confidence interval; eCVD, established cardiovascular disease; ICC, intraclass correlation coefficient; OR, odds ratio; QI, quality indicator.

| | QI 9 | | QI 10 | | QI 11 | |
|---|---|---|---|---|---|---|
| **Full model** | **OR (95 % CI)** | ***p*-value** | **OR (95 % CI)** | ***p*-value** | **OR (95 % CI)** | ***p*-value** |
| Intercept | 0.76 (0.52–1.09) | 0.14 | 0.39 (0.26–0.60) | < 0.001* | 6.94 (4.20–11.48) | < 0.001* |
| Diabetes | 1.43 (1.23–1.65) | < 0.001* | 1.71 (1.43–2.06) | < 0.001* | 0.82 (0.69– 0.97) | 0.02* |
| Hypertension | 3.39 (2.93–3.92) | < 0.001* | 0.97 (0.80–1.17) | 0.72 | 0.98 (0.82– 1.18) | 0.87 |
| eCVD | 1.36 (1.18–1.56) | < 0.001* | 3.39 (2.81–4.09) | < 0.001* | 0.98 (0.83– 1.16) | 0.82 |
| Male patient | 1.40 (1.23–1.59) | < 0.001* | 1.91 (1.62–2.25) | < 0.001* | 1.38 (1.17– 1.62) | < 0.001* |
| Patient age: 65–79 years (reference: < 65) | 1.57 (1.25–1.97) | < 0.001* | 1.37 (1.09–1.72) | 0.01* | 0.78 (0.57– 1.07) | 0.13 |
| Patient age: ≥ 80 years (reference: < 65) | 1.06 (0.85–1.32) | 0.62 | – | – | 1.22 (0.89– 1.68) | 0.22 |
| Male GP | 0.80 (0.62–1.04) | 0.10 | 0.82 (0.61–1.09) | 0.17 | 0.91 (0.65– 1.27) | 0.58 |
| GP age: 45–59 years (reference: < 45) | 1.16 (0.89–1.51) | 0.26 | 0.92 (0.69–1.23) | 0.58 | 0.85 (0.60– 1.18) | 0.33 |
| GP age: ≥ 60 years (reference: < 45) | 1.32 (0.90–1.92) | 0.15 | 1.08 (0.71–1.64) | 0.73 | 0.81 (0.51– 1.29) | 0.37 |
| Urban practice location | 1.01 (0.79–1.29) | 0.96 | 1.04 (0.79–1.36) | 0.78 | 0.83 (0.62– 1.12) | 0.22 |
| GP-level group variance, ICC | 0.25, 0.07 | | 0.20, 0.06 | | 0.26, 0.07 | |
| **Null model** | | | | | | |
| Intercept | 0.76 (0.57–1.01) | 0.06 | 0.31 (0.23–0.43) | < 0.001* | 5.62 (3.76–8.41) | < 0.001* |
| Diabetes | 1.45 (1.26–1.67) | < 0.001* | 1.75 (1.47–2.09) | < 0.001* | 0.82 (0.69–0.97) | 0.02* |
| Hypertension | 3.35 (2.92–3.84) | < 0.001* | 1.02 (0.85–1.22) | 0.85 | 0.99 (0.82–1.19) | 0.92 |
| eCVD | 1.36 (1.19–1.55) | < 0.001* | 3.46 (2.89–4.14) | < 0.001* | 0.98 (0.83–1.16) | 0.81 |
| Male patient | 1.36 (1.21–1.53) | < 0.001* | 1.90 (1.62–2.22) | < 0.001* | 1.37 (1.17–1.61) | < 0.001* |
| Patient age: 65–79 years (reference: < 65) | 1.55 (1.25–1.92) | < 0.001* | 1.36 (1.09–1.69) | 0.01* | 0.78 (0.57–1.07) | 0.12 |
| Patient age: ≥ 80 years (reference: < 65) | 1.05 (0.85–1.30) | 0.62 | – | – | 1.21 (0.88–1.67) | 0.23 |
| Urban practice location | 0.99 (0.79–1.26) | 0.96 | 1.07 (0.83–1.39) | 0.60 | 0.85 (0.63–1.13) | 0.26 |
| GP-level group variance, ICC | 0.25, 0.07 | | 0.21, 0.06 | | 0.26, 0.07 | |

*Statistically significant at level 0.05.
