# Supplementary material for: Quality and variation of care for chronic kidney disease in Swiss general practice: A retrospective database study
Source: PLoS One. 2022 Aug 11;17(8):e0272662. doi: 10.1371/journal.pone.0272662 (PMC9371276; doi:10.1371/journal.pone.0272662)
Supplement: S5 Table — “Full model” denotes the regression model including all predictors, while “Null model” denotes the model where demographic characteristics of general practitioners (GPs) were omitted. Abbreviations: CI, confidence interval; eCVD, established cardiovascular disease; ICC, intraclass correlation coefficient; OR, odds ratio; QI, quality indicator. (PDF) [file pone.0272662.s005.pdf]

S5 Table. Determinants of quality indicator achievement in the category *Treatment target achievement*. "Full model" denotes the regression model including all predictors, while "Null model" denotes the model where demographic characteristics of general practitioners (GPs) were omitted. Abbreviations: CI, confidence interval; eCVD, established cardiovascular disease; ICC, intraclass correlation coefficient; OR, odds ratio; QI, quality indicator.

	QI 12		QI 13		QI 14	
**Full model**	**OR (95 % CI)**	***p*-value**	**OR (95 % CI)**	***p*-value**	**OR (95 % CI)**	***p*-value**
Intercept	1.52 (1.22–1.90)	< 0.001*	1.09 (0.76–1.58)	0.63	0.32 (0.25–0.41)	< 0.001*
Diabetes	0.94 (0.87–1.03)	0.19	–	–	0.54 (0.48–0.60)	< 0.001*
Hypertension	0.72 (0.66–0.78)	< 0.001*	0.77 (0.65–0.91)	0.002*	0.82 (0.74–0.91)	< 0.001*
eCVD	1.16 (1.06–1.26)	0.001*	1.10 (0.93–1.29)	0.28	1.23 (1.10–1.37)	< 0.001*
Male patient	1.20 (1.12–1.30)	< 0.001*	1.16 (1.00–1.34)	0.05	0.96 (0.87–1.06)	0.45
Patient age: 65–79 years (reference: < 65)	0.79 (0.68–0.91)	0.001*	0.90 (0.70–1.14)	0.38	1.10 (0.91–1.33)	0.32
Patient age: ≥ 80 years (reference: < 65)	0.78 (0.68–0.90)	< 0.001*	0.85 (0.67–1.09)	0.19	1.97 (1.63–2.37)	< 0.001*
Male GP	1.01 (0.88–1.16)	0.91	1.17 (0.92–1.49)	0.19	1.05 (0.91–1.20)	0.53
GP age: 45–59 years (reference: < 45)	0.98 (0.85–1.13)	0.77	0.95 (0.75–1.21)	0.69	1.11 (0.97–1.27)	0.14
GP age: ≥ 60 years (reference: < 45)	1.00 (0.82–1.22)	0.99	0.93 (0.67–1.30)	0.69	1.01 (0.83–1.23)	0.91
Urban practice location	1.15 (1.00–1.33)	0.05*	1.26 (1.00–1.59)	0.05*	1.29 (1.13–1.48)	< 0.001*
GP-level group variance, ICC	0.19, 0.06		0.35, 0.10		0.06, 0.02	
**Null model**						
Intercept	1.48 (1.24–1.77)	< 0.001*	1.17 (0.87–1.58)	0.31	0.29 (0.24–0.36)	< 0.001*
Diabetes	0.95 (0.88–1.04)	0.26	–	–	0.80 (0.72–0.89)	< 0.001*
Hypertension	0.72 (0.66–0.78)	< 0.001*	0.76 (0.65–0.90)	0.001*	1.15 (1.04–1.28)	0.01*
eCVD	1.14 (1.05–1.25)	0.002*	1.05 (0.90–1.24)	0.52	0.94 (0.85–1.03)	0.18
Male patient	1.22 (1.14–1.32)	< 0.001*	1.19 (1.03–1.38)	0.02*	1.19 (0.99–1.43)	0.06
Patient age: 65–79 years (reference: < 65)	0.82 (0.71–0.94)	0.01*	0.91 (0.72–1.15)	0.44	2.21 (1.85–2.64)	< 0.001*
Patient age: ≥ 80 years (reference: < 65)	0.80 (0.69–0.92)	0.001*	0.87 (0.69–1.11)	0.27	1.26 (1.10–1.43)	< 0.001*
Urban practice location	1.12 (0.97–1.28)	0.12	1.25 (1.00–1.55)	0.05*	0.29 (0.24–0.36)	< 0.001*
GP-level group variance, ICC	0.20, 0.06		0.33, 0.09		0.07, 0.02	

*Statistically significant at level 0.05.
